# Magnetic dispersive solid-phase extraction of organochlorine pesticides from honey samples in a narrow-bore tube prior to HPLC analysis

**DOI:** 10.1039/d5ra01093d

**Published:** 2025-09-17

**Authors:** Mohammad Amin Rasoulizadeh, Mohammadhosein Movassaghghazani, Mohammad Reza Afshar Mogaddam

**Affiliations:** a Faculty of Veterinary Medicine, Shab. C., Islamic Azad University Shabestar Iran; b Department of Food Hygiene and Quality Control, Faculty of Veterinary Medicine, Shab. C., Islamic Azad University Shabestar Iran mh.movassagh@iau.ac.ir drmhmg@gmail.com; c Food and Drug Safety Research Center, Pharmaceutical Sciences Institute, Tabriz University of Medical Sciences Tabriz Iran; d Research Center of New Material and Green Chemistry, Khazar University 41 Mehseti Street Baku AZ1096 Azerbaijan; e Pharmaceutical Analysis Research Center, Pharmaceutical Sciences Institute, Tabriz University of Medical Sciences Tabriz Iran

## Abstract

The evaluation of pollutant residues, particularly pesticides, in honey samples is of utmost importance to maintain human safety. In this study, a magnetic dispersive solid-phase extraction method within a narrow-bore tube was introduced for the extraction and preconcentration of four organochlorine pesticides (OCPs) from honey samples. For this purpose, 40 mg of the synthesized magnetic Ni-MOF-I sorbent dispersed into 1 mL of acetonitrile was injected into the sample solution filled in a narrow-bore tube. An external magnet was placed near the end of the tube to collect the sorbent. After 5 min, the stopcock connected to the tube was opened, allowing the sample solution to pass through the sorbent held by the magnet at a flow rate of 5 mL min^−1^. The sorbent was then eluted using 250 μL of acetonitrile following the evaporation of the eluent. The analytes were redissolved in the mobile phase and analyzed by an analytical system. Under the optimized conditions, the analytes exhibited acceptable extraction recoveries ranging from 56% to 76% and a wide linear range of 1–1000 ng g^−1^ with an *r*^2^ value of ≥0.993. The limits of detection and quantification were found to be in the range of 0.11–0.25 ng g^−1^ and 0.37–0.84 ng g^−1^, respectively. Moreover, the proposed method demonstrated high precision with relative standard deviation values of ≤4.3% and 4.9% for intra- and inter-day precisions, respectively. Eventually, the desired approach was successfully carried out to monitor OCP residues in 30 honey samples, revealing no sign of the studied pesticides in any of the analyzed samples.

## Abstract 2

Development of a new sorbent based extraction procedure in narrow bore tube.

## Graphical abstract

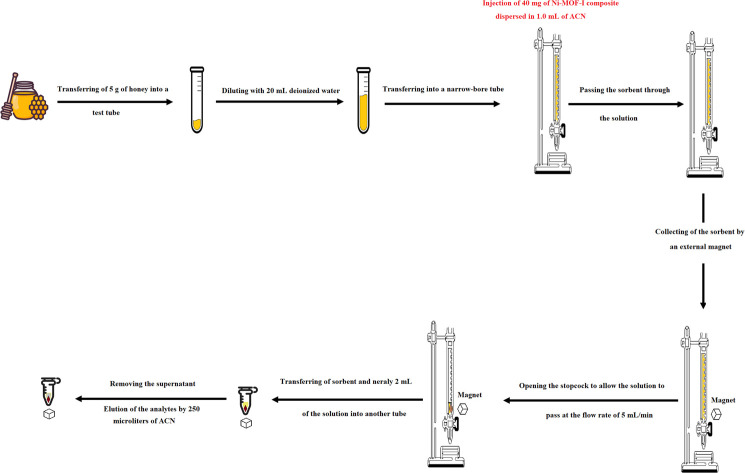

## 1 Introduction

Honey is a health-beneficial natural foodstuff known for its nutritional value and antimicrobial and antiseptic benefits.^1,2^ It is crucial for honey to be free from any chemical or biological contaminants to ensure its safety for human consumption. Indirect contamination can occur when pesticides are applied in agriculture, affecting the soil, air, water, and flowers.^3^ Additionally, pesticides can be imported into the food chain through honeybees, potentially leading to honey contamination.^4^ Pesticides are commonly used to control pests and enhance agricultural productivity, but their indiscriminate use can lead to the presence of pesticide residues in food, posing serious health risks to consumers, including cancers, birth defects, respiratory issues, and developmental delays.^5,6^ Organochlorine pesticides (OCPs), known as endocrine-disrupting chemicals, are persistent organic pollutants used in agriculture to control pests.^7^ The use of OCPs in agriculture has been forbidden due to their carcinogenic effects and potential to cause neurological dysfunction but they are still applied due to their effectiveness and cost-efficiency.^8^ Therefore, many countries have established maximum residue limits (MRLs) as a quality control measure. The MRL for most of the OCPs in honey samples is 0.01 mg kg^−1^, while the MRL for dichlorodiphenyltrichloroethane (DDT) and its metabolites is 0.05 mg kg^−1^.^9^ As a result, there is a growing demand for developing a rapid, sensitive, and reliable method to monitor pesticide residues in honey samples, especially to protect public health and global trade. Gas chromatography (GC) and high-performance liquid chromatography (HPLC) are outstanding analytical methods used for the determination of pesticide residues in honey.^10,11^ However, challenges such as matrix interference and trace amount of compounds can limit their effectiveness. Hence, employing an efficient sample preparation method is of great importance to obtain reliable results before their instrumental analysis.^12^ Solid-phase extraction (SPE) is a widely used technique in sample preparation for clean-up and analyte enrichment. It involves the use of a solid chromatographic packing material, typically in a cartridge or small column device, to extract target analytes.^13^ However, SPE has some drawbacks including the difficulty of simultaneous extractions, cartridge blockage, and long procedure times. To overcome these issues, a simplified variation of SPE called dispersive-SPE (DSPE) has been developed, in which the sorbent is not packed in a column but is directly dispersed into the sample solution containing the analytes, eliminating the need for a conditioning step.^14^ Unlike conventional SPE, which requires a constant and controlled sample flow rate to ensure proper interaction between the sorbent and target molecules, DSPE allows for immediate and more effective contact between the two phases.^15^ In recent years, magnetic nanomaterials have been chosen as the sorbent for DSPE, known as magnetic dispersive solid-phase extraction (MDSPE).^16^ An important benefit of MDSPE is that the magnetic nanoparticles (MNPs) can be easily separated from sample solutions using an external magnetic field, eliminating the need for extra centrifugation or filtration steps, which simplifies and accelerates the sample preparation procedure.^17^ Metal–organic frameworks (MOFs) represent a hybrid porous material, characterized by coordination polymers formed from organic ligands and metallic centers *via* coordination bonds.^18,19^ These materials offer a range of beneficial properties including high surface area, adsorption capacity, uniform cavities, thermal stability, adjustable porosity, structural flexibility, and customizable polarity. MOFs have found diverse applications in areas such as gas purification and separation, drug delivery, sample preparation techniques, gas storage, photovoltaic technology, catalysis, biomedicine, chromatographic column stationary phases, and sensing for spectroscopy and electroanalytical methods.^19–21^ Magnetic MOFs can be synthesized through various methods including hydrothermal, solvothermal, electrochemical, encapsulation, mechanochemical and sonochemical methods, as well as microwave-assisted synthesis, ball milling, and laser ablation. These methods affect the size, surface properties, crystallinity, shape, and ultimately, the magnetic behavior of the MOFs. The MOF enhances the stability of MNPs by protecting Fe_3_O_4_ nanoparticles from oxidation and degradation in different environments. Additionally, the organic linker of the MOF improves the surface stability of MNPs. Furthermore, MOFs can minimize the aggregation of MNPs and improve their dispersion in a matrix.^22,23^ The combination of MNPs with MOFs provides synergy between MNPs and MOFs, which can enhance the performance of magnetic MOFs in various aspects.^23^ The magnetization of MOFs enables easy separation and recycling in sample preparation methods without requiring centrifugation. Moreover, the catalytic properties of magnetic MOFs are enhanced by increased surface area, improved charge transfer, and synergistic interactions between the MOF and Fe_3_O_4_ components. Additionally, the inclusion of MOFs in the composite structure provides potential benefits such as high drug loading capacity and targeted delivery. However, the degradation of MOFs can result in the release of metal ions, which raises concerns regarding their long-term stability and biodegradability.

In this study, MDSPE in a narrow-bore tube was proposed for the simultaneous determination of four OCP residues in honey samples prior to HPLC-diode array detector (DAD) analysis. A magnetic composite of Ni-MOF-I was used as an environmentally friendly and cost-effective sorbent for the extraction and preconcentration of pesticides. This method does not require special equipment such as an autoclave and eliminates the use of organic solvents during the synthesis process. Ni-MOF-I is favored for the extraction of various compounds because of its high porosity, tunable pore size, and ability to interact with the analytes *via* hydrophobic interactions, hydrogen bonding, and π–π interactions between the MOF and the analyte. The method was performed in a narrow-bore tube allowing for the extraction of a relatively high volume of the sample to achieve sensitive detection limits. Performing the method using MNPs in a narrow tube enables easy collection after extraction with an external magnet, eliminating the need for centrifugation.

## 2 Experimental

### 2.1 Materials and solutions

The analytical grade standards of p,p-dichlorodiphenyldichloroethane (p,p′-DDD), p,p′-dichlorodiphenyldichloroethylene (p,p′-DDE), p,p′-dichlorodiphenyltrichloroethane (p,p′-DDT) and o,p′-DDT with a purity > 98% were purchased from Dr Ehrenstorfer (Augsburg, Germany). The solid materials including iron(ii) sulfate heptahydrate (FeSO_4_·7H_2_O), iron(iii) chloride hexahydrate (FeCl_3_·6H_2_O), nickel(ii) chloride hexahydrate (NiCl_2_·6H_2_O), 1,4-benzenedicarboxylic acid (1,4-BDCA), and sodium chloride (NaCl) were acquired from Sigma-Aldrich (St. Louis, MO, USA). An ammonia solution (25%, w/w), acetonitrile (ACN), acetone, methanol, and iso-propanol were obtained from Merck (Darmstadt, Germany). Deionized water was procured from Ghazi Co. (Tabriz, Iran). A mixture stock solution of OCPs at a concentration of 50 mg L^−1^ of each analyte was prepared in methanol. The working standard solutions were prepared daily by diluting the stock solution with deionized water at required concentrations.

### 2.2 Real samples

Thirty honey samples were obtained from local markets in Tabriz, Iran. Additionally, a honey sample from a producer located far away from agricultural regions, known to be free from pesticides, was utilized as the blank sample for optimizing and validating the extraction procedure.

### 2.3 Instruments

The chromatographic analysis was carried out using an Agilent HPLC (Model 1200) equipped with a DAD. The separation of the studied analytes was done using a ZORBAX C_18_ column (100 mm × 4.6 mm i.d., and 5 μm particle size) adjusted at 40 °C. Isocratic elution was performed using a water:methanol (30 : 70, v/v) mixture as the mobile phase at a flow rate of 1 mL min^−1^. The absorption wavelength for the determination of all OCPs was 238 nm.

### 2.4 Preparation of the magnetic composite of Ni-MOF-I

The synthesis method was performed according to the literature.^24^ For the preparation of Ni-MOF-I, according to the previous report, NiCl_2_·6H_2_O as a cluster coordinated with 1,4-BDCA as a ligand. For this, initially, 0.80 g of 1,4-BDCA was combined with 5 mL of concentrated ammonia solution under stirring. Subsequently, 20 mL of deionized water was added dropwise until the solution clarified. The resulting solution (I) was then transferred to a burette.

Afterwards, 1.14 g of NiCl_2_·6H_2_O was dissolved in 100 mL deionized water taken in an Erlenmeyer flask (II), which was then placed in a water bath adjusted at 80 °C.

Under stirring, solution (I) was added dropwise to solution (II). After that, the obtained mixture was stirred for 1 h. Following this, the mixture was cooled down to room temperature, leading to the formation of a light green precipitate. The solution was then transferred to multiple 10 mL glass test tubes to separate Ni-MOF-I formed by centrifugation at 6000 rpm for 5 min, followed by discarding the supernatant. The precipitate was eluted with deionized water (5 mL × 5) and finally dried overnight at room temperature.

Subsequently, for the preparation of the magnetic composite of Ni-MOF-I MOF (X/Y 1/3) (X = Ni-MOF-I and Y = Fe_3_O_4_), 187.5 mg of the synthesized Ni-MOF-I was added to 40 mL deionized water and subjected to sonication for half an hour to remove dissolved oxygen. Then, FeSO_4_·7H_2_O (0.9 g) and FeCl_3_·6H_2_O (1.325 g) were added to the above solution, followed by heating in a water bath at 80 °C with continuous stirring. Then, 12 mL concentrated ammonia solution was added dropwise under stirring. After the completion of the addition, the mixture was kept under stirring for 2 h. Eventually, the resulting composite was collected using a magnet and washed with a mixture of water:ethanol (50 : 50% v/v) for five times. The composite was then dried at 60 °C for 6 h.

### 2.5 MDSPE procedure

In the first step, 5 g of blank honey sample spiked with analytes (50 ng g^−1^, of each analyte) was diluted with 20 mL deionized water and transferred into a narrow bore glass tube (100 cm × 0.5 cm i.d.) with a funnel-like head, where the end of the tube was narrower and a stopcock was connected to it. Next, 40 mg of the magnetic composite of Ni-MOF-I, along with 1 mL of ACN, was added to the tube using a glass syringe to disperse the sorbent into the sample solution. The sorbent particles moved down due to gravity and were collected at the end of the tube using an external magnetic field. After 5 min, the sample was passed through the sorbent particles at a speed of 5 mL min^−1^ in the presence of a magnet by opening the stopcock. Towards the end (after at least 18 mL of the aqueous phase had passed through), the external magnetic field was removed, and the final 2 mL of solution, containing both the sorbent and analytes, was collected in a 3 mL test tube. The aqueous phase was then removed in the presence of a magnet. Subsequently, 250 μL of ACN was added as an eluent to the sorbent particles for the desorption of the analytes and vortexed for 5 min. Finally, in the presence of a magnet, after transferring the organic phase to a microtube and evaporating it in a nitrogen atmosphere, the residue was dissolved in 50 μL of the mobile phase and injected into the HPLC-DAD for analysis. The method steps are shown in Fig. S1.

## 3 Results and discussion

### 3.1 Optimization of effective parameters

#### 3.1.1 Selection of sorbent type and amount

The efficiency of the developed method is related to the type of sorbent used. In order to demonstrate the synergistic effect of Fe_3_O_4_ and Ni-MOF-I, a series of experiments were conducted using both bare Fe_3_O_4_ and the prepared composite. In each experiment, 50 mg of each compound was utilized for extracting the analytes from the sample solution. The results, shown in Fig. S2, indicated that while bare Fe_3_O_4_ could adsorb analytes, the formation of the composite significantly enhanced the efficiency of the method. This improvement can be attributed to the high porosity and larger contact area of the composite compared to bare Fe_3_O_4_. As a result, the magnetic Ni-MOF-I composite was selected for use in the next tests.

**Fig. 1:**
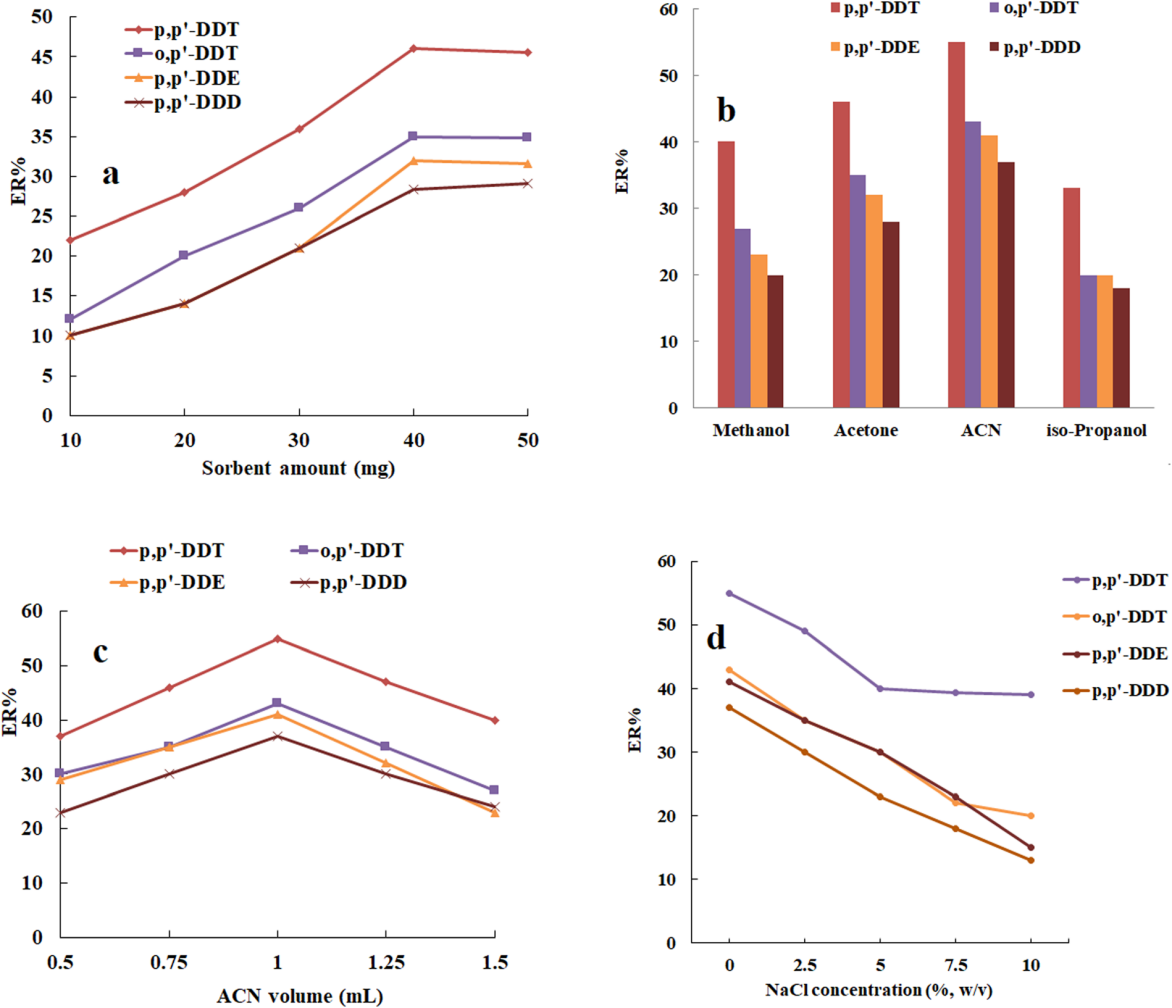
Optimization of the (a) magnetic sorbent amount, (b) dispersive solvent type, (c) dispersive solvent volume, and (d) ionic strength. (a) Conditions: sample, 5 g honey sample; dispersive solvent (volume): acetone (1 mL); extraction time: 5 min; flow rate: 5 mL min^−1^; desorption solvent (volume): methanol (200 μL); and agitation type (time) in desorption step: vortexing (5 min). (b) Conditions: the same as those utilized in (a), except that 40 mg of Ni-MOF-I was used as the sorbent amount. (c) Conditions: the same as those utilized in (b), except that ACN was selected as the dispersive solvent type. (d) Conditions: the same as those utilized in (c), except that 1 mL of ACN was opted as the dispersive solvent volume.

The sorbent amount is a crucial parameter influencing the clean-up process in the sorbent-based methods. The amount of magnetic composite of Ni-MOF-I was tested in the range of 10–50 mg to evaluate their effect on ERs of the analytes. As indicated in Fig. 1a, the ERs of the analytes improved up to 40 mg due to the increased access to adsorption sites. However, lower ERs at higher amounts likely attributed to the incomplete elution of analytes from the sorbent surface. Therefore, 40 mg was selected for subsequent steps.

#### 3.1.2 Optimization of the dispersive solvent type and its volume

In magnetic dispersive solid-phase extraction in a narrow-bore tube, the consumption of a dispersive solvent is unavoidable to disperse the sorbent into the sample solution. To acquire the highest analytical signal, the efficiencies of iso-propanol, ACN, acetone, and methanol were tested (1 mL of each, separately). According to the Fig. 1b, ACN indicates a better performance in the dispersion of the selected sorbent. Therefore, ACN was chosen as the optimum dispersive solvent type for the following steps.

After the optimization of the dispersive solvent type, the effect of its volume on ERs of the target analytes was evaluated using different ACN volumes (0.5–1.5 mL). Fig. 1c shows that 1 mL ACN results in the highest ERs and it was applied as the optimum dispersive solvent volume in this work.

#### 3.1.3 Optimization of the NaCl concentration

The extraction recoveries (ERs) of the target analytes in the MDSPE method can be altered by incorporating NaCl, which affects the extraction procedure through salting-out and salting-in effects.^26^ Salting-out involves reducing the solubility of analytes in the sample solution, resulting in higher ERs by increasing the ionic strength of the aqueous phase. Conversely, salting-in, induced by NaCl addition, increases the viscosity of the aqueous phase, negatively impacting the extraction efficiency by decreasing the diffusion coefficients of the targeted analytes. Therefore, the impact of ionic strength on ERs of the analytes was investigated by dissolving different NaCl concentrations in the range of 0% to 10% w/v in the sample solution. According to the findings in Fig. 1d, The ER values of the analytes decreased as the NaCl concentration increased, primarily due to the salting-in effect. Consequently, subsequent studies were conducted in the absence of NaCl.

#### 3.1.4 Assessment of the flow rate

**Fig. 2:**
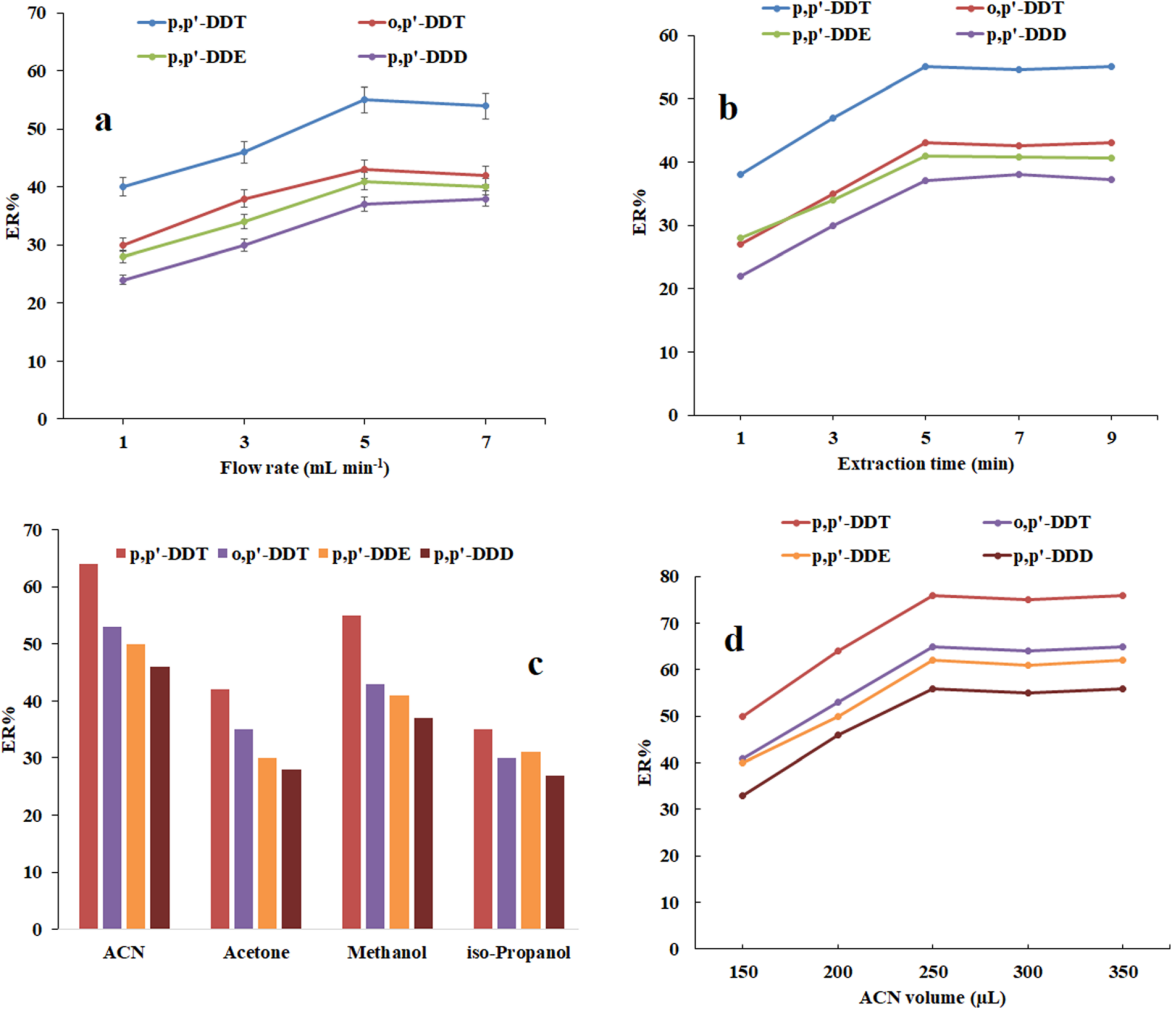
Optimization of (a) flow rate, (b) extraction time, (c) elution solvent type, and (d) eluent volume. (a) Conditions: the same as those utilized in Fig. 1d, except that the extraction procedure was done in the absence of NaCl. (b) Conditions: the same as those utilized in (a), except that 5 mL min^−1^ was selected as the optimal flow rate. (c) Conditions: the same as those utilized in (b), except that 5 min was selected as the extraction time. (d) Conditions: the same as those utilized in (c), except that ACN was opted as elution solvent.

The rate of passing the sample solution through the sorbent bed is a critical factor affecting both ERs of the OCPs and extraction time.^25^ To determine the optimal flow rate in the magnetic dispersive solid-phase extraction in a narrow-bore tube, several tests were conducted at a flow rate ranging from 1 to 7 mL min^−1^, as depicted in Fig. 2a. The results showed that the highest analytical signals were obtained when the sample solution passed at a flow rate of 5 mL min^−1^ through the bed of the sorbent. This is attributed to the sufficient time available for the sorbent to interact with the analytes and efficiently extract them from the sample matrix. Consequently, 5 mL min^−1^ was selected as the optimum flow rate for subsequent experiments.

#### 3.1.5 Assessment of the extraction time

To ensure the optimal interaction between the studied analytes and sorbent particles, sufficient extraction time is needed to acquire the highest ERs. In the present study, after adding the sorbent particles to the sample solution, the time between adding the sorbent to the sample and opening the stopcock is defined as the extraction time. To investigate the optimal extraction time, a series of experiments were conducted in the range of 1–7 min. The findings presented in Fig. 2b show that 5 min is sufficient to migrate analytes through the sample solution onto the sorbent surface and more than that had no significant effect on the ERs of the analytes. Therefore, 5 min was selected as the optimal extraction time for subsequent experiments.

#### 3.1.6 Optimization of the eluent type and its volume

A crucial aspect of selecting a proper eluent is its effectiveness in desorbing analytes from the surface of the sorbent. According to the polarity of OCPs, water-miscible organic solvents including iso-propanol, ACN, acetone, and methanol were considered for this purpose. Based on the outcomes presented in Fig. 2c, ACN was identified as the suitable elution solvent and chosen for the following studies.

The volume of the eluent plays a crucial role in affecting the ER of pesticides. To obtain the optimal volume of the extraction solvent, the offered method was conducted within the range of 150–350 μL of ACN. Based on the obtained results in Fig. 2d, the analytical signals of all OCPs increase up to 250 μL and then almost remain constant. As a result, 250 μL was identified as the optimal eluent volume in this study.

#### 3.1.7 Optimization of the agitation type and time

**Fig. 3:**
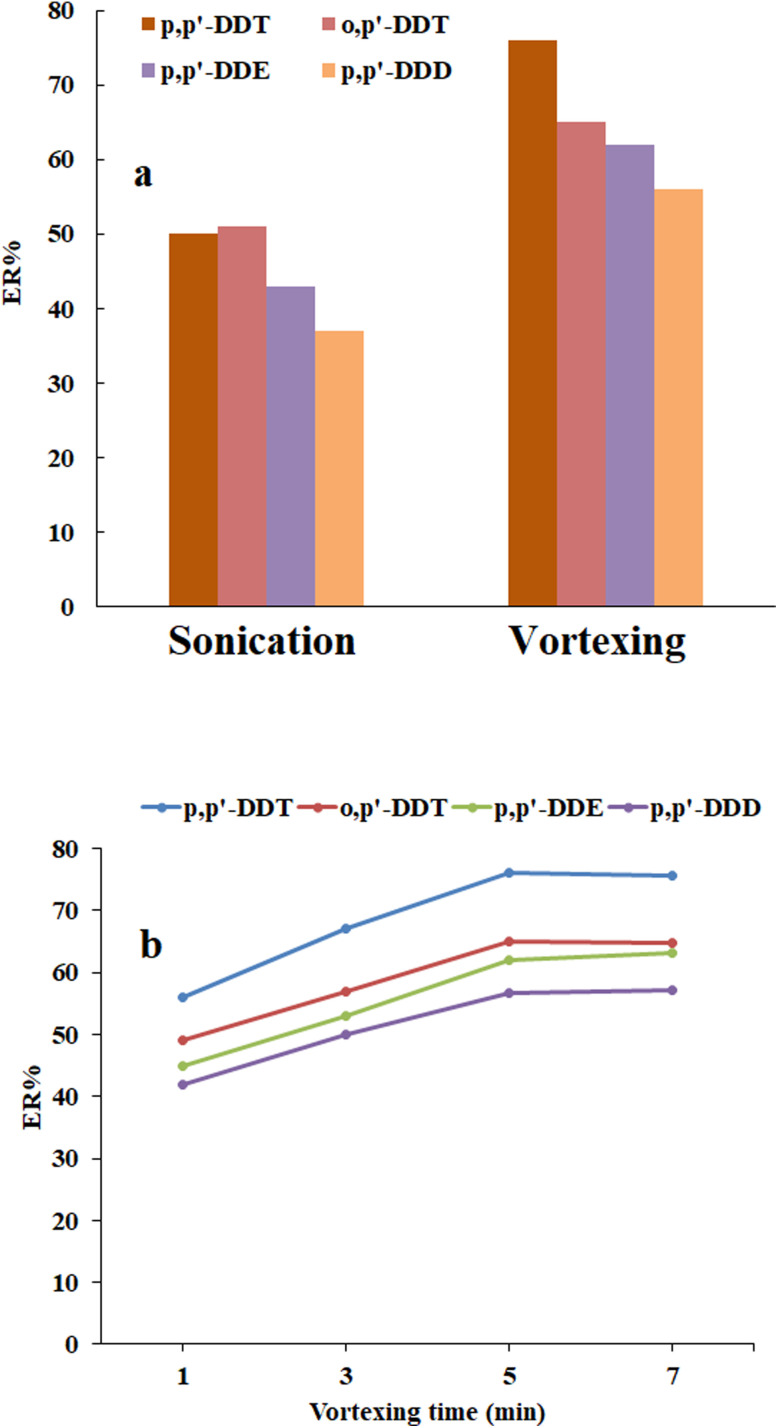
Optimization of (a) agitation mode and (b) its time. (a) Conditions: the same as those used in Fig. 2d, but the eluent volume was 250 μL. (b) Conditions: the same as those used in (a), but the vortexing time was 5 min.

To improve the contact area of the elution solvent with the sorbent during the desorption step, the influence of agitation mode was examined using both vortexing and sonication. The results presented in Fig. 3a indicated that the highest ERs of the analytes were obtained when employing vortex agitation, making it the preferable desorption mode.

In order to assess the impact of desorption time on method efficiency, a mixture of sample solution containing the analytes and the sorbent was vortexed within a range of 1 to 9 min. The obtained results illustrated in Fig. 3b show that 5 min was sufficient for achieving the maximum desorption efficiency. Consequently, subsequent experiments were conducted using 5 min as the preferred desorption time.

### 3.2 Stability of the sorbent

The capability of the composite prepared from Fe_3_O_4_ and Ni-MOF-I for repeated extraction of analytes was investigated under optimal conditions. The data depicted that there was no memory effect, confirming the complete desorption of analytes from the sorbent surface in the first extraction procedure. Furthermore, the results demonstrated that the efficiency of the method remains consistent after using the same sorbent five times with relative standard deviation (RSD) values ≤ 6.0%.

### 3.3 Method validation

The feasibility of the suggested procedure was validated considering key parameters such as linear range (LR), coefficient of determination (*r*^2^), relative standard deviation (RSD), limits of detection (LODs, signal-to-noise ratio of 3) and quantification (LOQs, signal-to-noise ratio of 10), and ER. As shown in Table 1, the calibration curves of OCPs demonstrated board linearity within the range of 1–1000 ng g^−1^ with *r*^2^ higher than 0.993. The precisions of the offered method, expressed as RSD values, were evaluated at 10 ng g^−1^. The intra-day precisions ranged from 3.1 to 4.3% (*n* = 6), while inter-day precisions were found to be in the range of 3.9–4.9%. The LOD and LOQ values were determined to be in the ranges of 0.11–0.25 and 0.37–0.84 ng g^−1^, respectively. The ER was calculated according to the following equation:

1 ER% = (*V*_eluent_/*V*_h_) × *C*_eluent_/*C*_h_ × 100

where *V*_h_ is the volume of honey, *V*_eluent_ is the volume of the eluent used for the desorption of analytes, *C*_eluent_ is the analyte concentration in the eluent, and *C*_h_ is the concentration in honey. The ER values for OCPs were obtained in the range of 56 to 76%.

**Table 1:** Figures of merit of the offered method

Analyte	LOD^a^	LOQ^b^	LR^c^	*r* ^2^ d	RSD^e^ %		ER ± SD^f^
					Intra-day	Inter-day	
p,p′-DDT	0.11	0.37	1–1000	0.995	4.3	4.9	76 ± 2
o,p′-DDT	0.25	0.84	1–1000	0.998	3.9	4.3	65 ± 4
p,p′-DDE	0.14	0.47	1–1000	0.993	3.5	4.3	62 ± 5
p,p′-DDD	0.23	0.77	1–1000	0.997	3.1	3.9	56 ± 2

a Limit of detection (S/N = 3) (ng g^−1^).

b Limit of quantification (S/N = 10) (ng g^−1^).

c Linear range (ng g^−1^).

d Coefficient of determination.

e Relative standard deviation for intra-day (*n* = 6) and inter-day (*n* = 4) precisions at a concentration of 10 ng g^−1^ of each analyte.

f Extraction recovery ± standard deviation (*n* = 3).

### 3.4 Analysis of honey samples

To assess the application of the proposed approach in the determination of OCP residues in real samples, the MDSPE-HPLC procedure was employed for the monitoring of p,p′-DDT, o,p′-DDT, p,p′-DDE and p,p′-DDD in thirty honey samples, under the optimum conditions. Based on the outcomes, the OCP levels in all studied samples were under the LOQ.

To evaluate the matrix effect, five honey samples were randomly selected and they were spiked with OCPs at three concentrations (5, 25, and 100 ng g^−1^). These samples were extracted and then analyzed by the method. The results obtained for the pesticides in the honey samples were compared to those from the blank sample spiked at corresponding concentrations and expressed as mean relative recovery (RR%). With respect to the outcomes presented in Table 2, the findings (RR between 82% and 102%), indicated that the sample matrices have a negligible impact on the performance of the developed method.

**Table 2:** Results of the matrix effect study

Analyte	Mean relative recovery ± standard deviation (*n* = 3)				
	Sample 1	Sample 2	Sample 3	Sample 4	Sample 5
**All samples were spiked with each analyte at a concentration of 5 ng g** ^**−1**^					
p,p′-DDT	83 ± 2	89 ± 2	95 ± 5	94 ± 3	98 ± 5
o,p′-DDT	82 ± 5	86 ± 2	95 ± 5	93 ± 2	95 ± 4
p,p′-DDE	87 ± 3	92 ± 4	90 ± 4	90 ± 2	92 ± 2
p,p′-DDD	92 ± 2	94 ± 5	89 ± 2	90 ± 5	94 ± 2

**All samples were spiked with each analyte at a concentration of 25 ng g** ^**−1**^					
p,p′-DDT	87 ± 2	95 ± 1	98 ± 3	94 ± 2	101 ± 6
o,p′-DDT	89 ± 2	92 ± 4	99 ± 5	97 ± 5	98 ± 2
p,p′-DDE	94 ± 4	97 ± 3	96 ± 4	94 ± 2	96 ± 5
p,p′-DDD	97 ± 5	98 ± 5	95 ± 4	96 ± 3	98 ± 4

**All samples were spiked with each analyte at a concentration of 100 ng g** ^**−1**^					
p,p′-DDT	94 ± 2	99 ± 4	102 ± 5	96 ± 3	100 ± 4
o,p′-DDT	93 ± 4	97 ± 2	102 ± 6	99 ± 2	102 ± 6
p,p′-DDE	98 ± 4	100 ± 6	99 ± 2	97 ± 5	94 ± 2
p,p′-DDD	99 ± 5	102 ± 4	98 ± 4	100 ± 4	96 ± 2

### 3.5 Comparison

To demonstrate the effectiveness of the method for extracting and analyzing specific pesticides in various samples, certain analytical parameters such as LR, LOD, ER, and EF are compared with those from previously published studies.^27–29^ The findings are outlined in Table S2. This approach exhibits broad linear ranges and LODs that are either lower or similar to those of the majority of the methods referenced. Furthermore, the EFs achieved with this method are superior to those of the methods documented in the literature (Table 3).

**Table 3:** Comparison of the proposed method with other approaches for the determination of OCPs

Analyte	Method	Sample	LOD^a^	LOQ^b^	LR^c^	RSD^d^ %	Reference
4,4′-DDT	DSPE-GC-ECD^e^	Environmental water	0.22	0.73	1–2000	5.7–6.8	27
4,4′-DDE	SB-μ-SPE-GC-MS^f^	Environmental water	0.33	—	1–100	8.9–9.6	28
4,4′-DDD			0.25	—	1–100	10.9–11.5	
4,4′-DDT			0.34	—	1–100	7.5–8.2	
p,p′-DDT	MSPE-GC-MS/MS^g^	Agricultural irrigation water	1.03	—	2–200	3.1	29
o,p′-DDT			0.74	—	2–200	3.9	
p,p′-DDE			0.45	—	1–200	1.3	
p,p′-DDD			0.41	—	2–200	3.1	
p,p′-DDT	MDSPE-HPLC-DAD^h^	Honey samples	0.11	0.37	1–1000	4.3–4.9	This work
o,p′-DDT			0.25	0.84	1–1000	3.9–4.3	
p,p′-DDE			0.14	0.47	1–1000	3.5–4.3	
p,p′-DDD			0.23	0.77	1–1000	3.1–3.9	

a Limit of detection (ng g^−1^).

b Limit of quantification (ng g^−1^).

c Linear range (ng g^−1^).

d Relative standard deviation.

e Dispersive solid-phase extraction-gas chromatography-electron capture detector.

f Stir-bar supported membrane protected micro-solid-phase extraction-gas chromatography-mass spectrometry.

g Magnetic solid-phase extraction-gas chromatography-tandem triple quadrupole mass spectrometry.

h Ultrasonic assisted liquid–liquid microextraction-gas chromatography-mass spectrometry.

## 4 Conclusions

Herein, a MDSPE-HPLC-DAD approach for the extraction and monitoring of widely used OCPs from thirty honey samples in a narrow-bore tube was reported. In the present study, a magnetic composite of Ni-MOF-I was used as an efficient sorbent using a green synthesis process. The developed technique demonstrated favorable analytical performance, including low LOD and LOQ values, good repeatability of 71–83% with RSD values ≤ 4.9%, wide LRs, and tolerable ER values. The major aspect of the proposed method is the synthesis of the sorbent in an aqueous medium and elimination of the time-consuming centrifugation steps. Additionally, no significant matrix effect was observed in the analysis of honey samples. Based on the compelling evidence and explanations provided, it can be inferred that the developed approach is rapid, sensitive and reliable for the extraction and simultaneous determination of trace levels of the OCPs in various food samples.

## Conflicts of interest

There are no conflicts to declare.

## Abbreviations

OCP: Organochlorine pesticide
MDSPE: Magnetic dispersive solid-phase extraction
HPLC: High-performance liquid chromatography
DAD: Diode array detector
MOF: Metal–organic framework

## Supplementary Material

RA-015-D5RA01093D-s001

## Data Availability

Supplementary information: several results such as figures. See DOI: https://doi.org/10.1039/d5ra01093d.
