# Supplementary material for: Magnetic dispersive solid-phase extraction of organochlorine pesticides from honey samples in a narrow-bore tube prior to HPLC analysis
Source: RSC Adv. 2025 Sep 17;15(40):33896–904. doi: 10.1039/d5ra01093d (PMC12442027; doi:10.1039/d5ra01093d)
Supplement: RA-015-D5RA01093D-s001 [file RA-015-D5RA01093D-s001.pdf]

Supplementary Information (SI) for RSC Advances.
This journal is © The Royal Society of Chemistry 2025

Development of a magnetic dispersive solid phase extraction in a narrow-bore tube for the extraction of organochlorine pesticides in honey samples prior to HPLC analysis

Mohammad Amin Rasoulizadeh[1], Mohammadhosein Movassaghghazani[2,*], Mohammad Reza Afshar Mogaddam [3,4,5]

[1] *Faculty of Veterinary Medicine, Shab.C., Islamic Azad University, Shabestar, Iran*

[2] *Department of Food Hygiene and Quality Control, Faculty of Veterinary Medicine, Shab.C., Islamic Azad University, Shabestar, Iran,*

[3] *Food and Drug Safety Research Center, Pharmaceutical Sciences Institute, Tabriz University of Medical Sciences, Tabriz, Iran*

[4] *Research Center of New Material and Green Chemistry, Khazar University, 41 Mehseti Street, Baku AZ1096, Azerbaijan*

[5]*Pharmaceutical Analysis Research Center, Pharmaceutical Sciences Institute, Tabriz University of Medical Sciences, Tabriz, Iran*

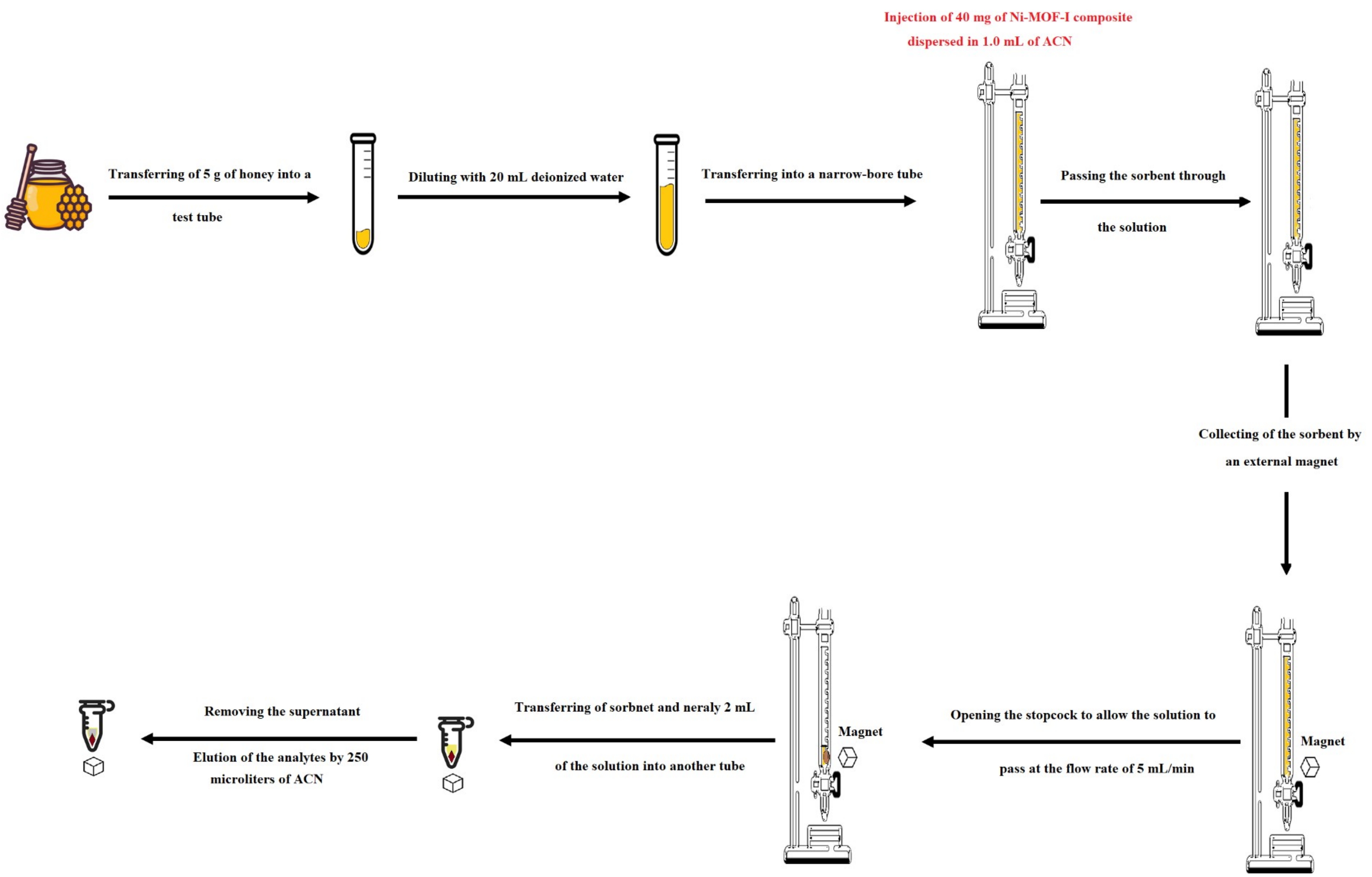

Fig. S1. The extraction method steps.

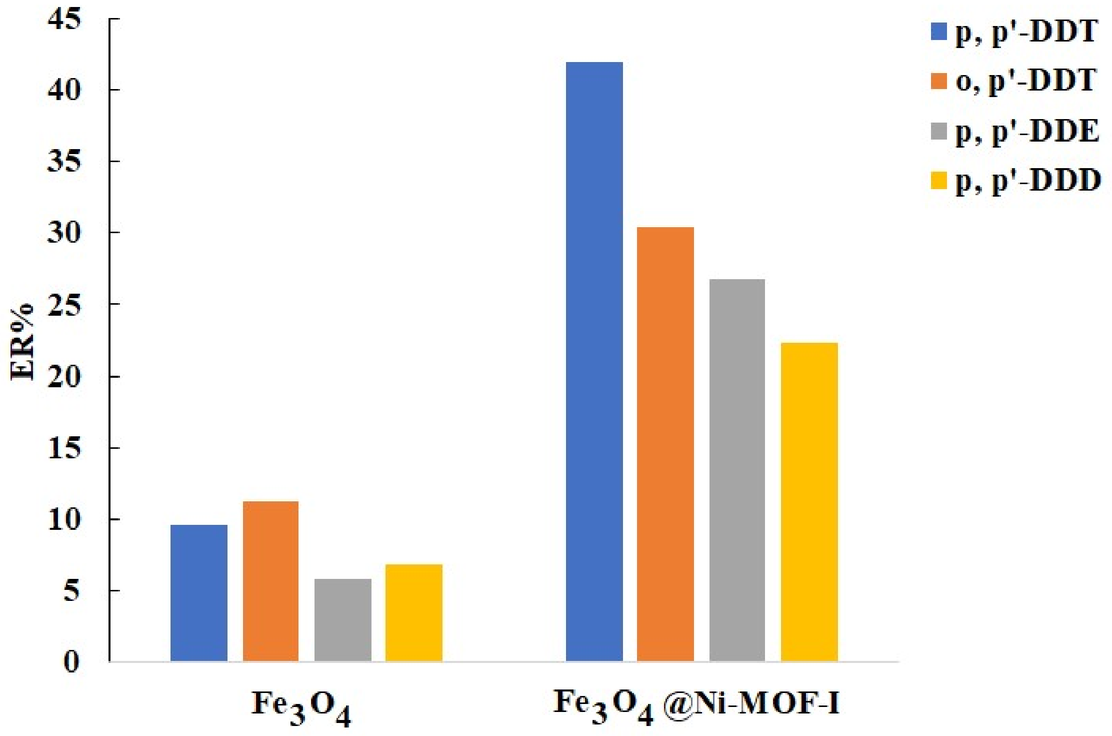

Fig. S2. Selection of adsorbent type.

(a) Conditions: sample, 5 g honey sample spiked with 50 ng g^{-1} of each analyte; dispersive solvent (volume): acetone (1 mL); sorbent amount: 50 mg; extraction time: 5 min; flow rate: 5 mL min^{-1}; desorption solvent (volume): methanol (200 µL); and agitation type (time) in desorption step: vortexing (5 min).
